# Motion Sickness, Binocular Visual Functions, and Visual Perception

**DOI:** 10.3390/jcm15041529

**Published:** 2026-02-15

**Authors:** Ching-Ying Cheng, Hung-Rui Chen, Po-Yu Chen, Lung-Hui Tsai, Tun-Shin Lo, Chi-Wu Chang

**Affiliations:** 1 Department of Optometry, Chung Shan Medical University, Taichung 402, Taiwan; ldiioul.tw@gmail.com (C.-Y.C.); chenicktw@gmail.com (H.-R.C.); x5217521742@gmail.com (P.-Y.C.); a0921583364@gmail.com (L.-H.T.); 2 Department of Ophthalmology, Chung Shan Medical University Hospital, Taichung 402, Taiwan; 3 Department of Speech Language Pathology and Audiology, Chung Shan Medical University, Taichung 402, Taiwan; 4 Department of Otolaryngology, Chung Shan Medical University Hospital, Taichung 402, Taiwan

**Keywords:** binocular visual function, visual perception, motion sickness susceptibility

## Abstract

**Clinical Relevance and Background:** Motion sickness is a common manifestation of autonomic dysfunction. Increasingly induced by modern technology, such as virtual reality (VR), it presents a pressing challenge that warrants investigation. However, the relationship between binocular function, visual perception, and motion sickness remains largely unexplored. Therefore, this study investigated the correlations between binocular visual functions, visual perception, and motion sickness susceptibility in adults. **Methods:** Adults aged 20 to 25 years were recruited. Based on a background and motion sickness susceptibility questionnaire, participants were divided into two groups: the Sick Tendency (ST) group (n = 21) and the Normal group (n = 33). Clinical assessments included habitual distance prescription and visual acuity (VA), phoria, fixation disparity (FD), positive/negative fusional vergence (PFV/NFV), vertical fusional vergence (VFV), positive/negative relative accommodation (PRA/NRA), accommodative facility (AF), vergence facility (VF), stereopsis, contrast sensitivity (CS), near point of convergence (NPC), and near point of accommodation (NPA). Additionally, motor-free visual perception test (MVPT), peripheral awareness (PA), and body balance (center of pressure) were assessed. **Results:** The ST group exhibited significantly higher distance NFV, distance VFV, and near PFV. Conversely, their NPA, stereopsis, and body balance (center of pressure) were significantly poorer than those of the Normal group. These deficits may be attributed to the accommodation–convergence conflict. **Conclusions:** Motion sickness susceptibility is closely associated with specific binocular functions. Individuals susceptible to motion sickness exhibit poorer postural stability, likely due to diminished stereopsis and accommodative amplitude (NPA). Future research should further investigate the underlying mechanisms and their clinical implications.

## 1. Introduction

Motion sickness susceptibility is a common phenomenon experienced by individuals in daily life; however, the threshold for experiencing dizziness varies among individuals. In the United States, more than one-third of the population has experienced motion sickness and shown symptoms such as nausea, vomiting, and sweating [1].

According to the prevailing theory regarding motion sickness, its onset is attributed to sensory conflict arising during sensory integration [2,3]. Three types of sensory input are related to motion sickness: visual sensory input, vestibular sensory input, and proprioceptive sensory input [2,3,4,5]. With the coordination of these three sensory systems, individuals can systematically and efficiently maintain their balance. However, if one of these sensory inputs encounters issues during signal processing, it may cause a decline in balance ability [3] or prompt the reliance on other sensory inputs for compensation to maintain equilibrium [5,6]. Sensory conflict signals are transmitted to the brain’s vomiting center, which includes the cerebellum, the medulla oblongata, and the reticular formation. The nerves responsible for conveying these vomiting responses include the eighth cranial nerve (vestibulocochlear nerve), the tenth cranial nerve (vagus nerve), and the nucleus tractus solitarius. Subsequently, these signals are transmitted to the gastrointestinal tract; ultimately, they trigger vomiting [7].

Sensory conflict can be categorized into two common types. The first type occurs because of a contradiction between visual information and vestibular information. The second type results from inconsistent signals between the semicircular canals and the otolith organs within the vestibular system [8]. Furthermore, visual, proprioceptive, and vestibular senses influence the center of gravity and postural sway; among them, vision is the most dominant factor [3]. In previous studies, vestibular and visual stimuli are used to assess motion sickness susceptibility and its associated performance [9,10]. If a person exhibits considerable susceptibility to motion sickness, it may be attributed to the interaction between genetic and environmental factors [11,12,13]. Some studies have indicated that women are more likely to experience motion sickness than age-matched men are and often report more severe symptoms, particularly during their menstrual periods [14,15,16]. Other studies have provided evidence that pregnant women are especially susceptible to motion sickness, possibly because of hormonal changes during pregnancy [7,13]. Children under the age of 2 years rarely experience motion sickness or exhibit symptoms, likely because they lack sufficient visual input signals [16,17]. Conversely, children aged 6–12 years are the most affected by motion sickness, peaking between the ages of 9 and 10 years [13,14,15,18].

Few studies have investigated several factors, including visual-spatial abilities in visual perception, which may reduce motion sickness susceptibility [17]. However, binocular visual function has rarely been discussed in this context. In natural conditions, humans unconsciously maintain posture, a phenomenon known as postural sway. It is defined as the horizontal oscillation around an individual’s center of gravity [19]. This term also covers one’s ability to maintain their body while standing still. Increased postural sway indicates crucial body movements during standing, which is often regarded as a potential indicator of various conditions or diseases, such as natural aging, neuromuscular disorders, anxiety, or attention deficit hyperactivity disorder [19,20,21]. Based on this understanding, the primary motivation of this study was to explore the relationship between motion sickness susceptibility, binocular visual function, visual perception, and balance ability.

## 2. Materials and Methods

### 2.1. Study Design

This experimental study was approved by the Institutional Review Board of Human Experimentation Committee of Chung Shan Medical University Affiliated Hospital. All participating researchers were equipped with good clinical practice education and training certificates for human trials and were strictly adherent to the ethical principles of the Helsinki Declaration. The study was conducted at the specialized optometry laboratory of the Department of Optometry at Chung Shan Medical University.

### 2.2. Research Subjects

Healthy young adults aged 20–25 years, with best corrected visual acuity ≥ 0.8 at both distance and near, were recruited. All the participants were primarily composed of students from Chung Shan Medical University. The participants who consented to join the study were asked to sign an informed consent form and complete a motion sickness susceptibility questionnaire (MSSQ), along with visual acuity measurements. Individuals with relevant ocular diseases, long-term visual disturbances, or conditions affecting balance were excluded. The participants had the right to withdraw from the study at any time. The sample size for the current study was determined using G*Power 3.1 based on an independent samples *t*-test (two-tailed). With an effect size of d = 0.80, α = 0.05, and power (1 − β) = 0.8, the required total sample size was 52. Our actual sample size of 54 participants fulfilled this requirement, providing a post hoc power of 0.82. It should be noted that the more complex repeated measures design involving within-between interactions is reserved for the second stage of this research project, which will further investigate longitudinal changes.

**Figure 1:**
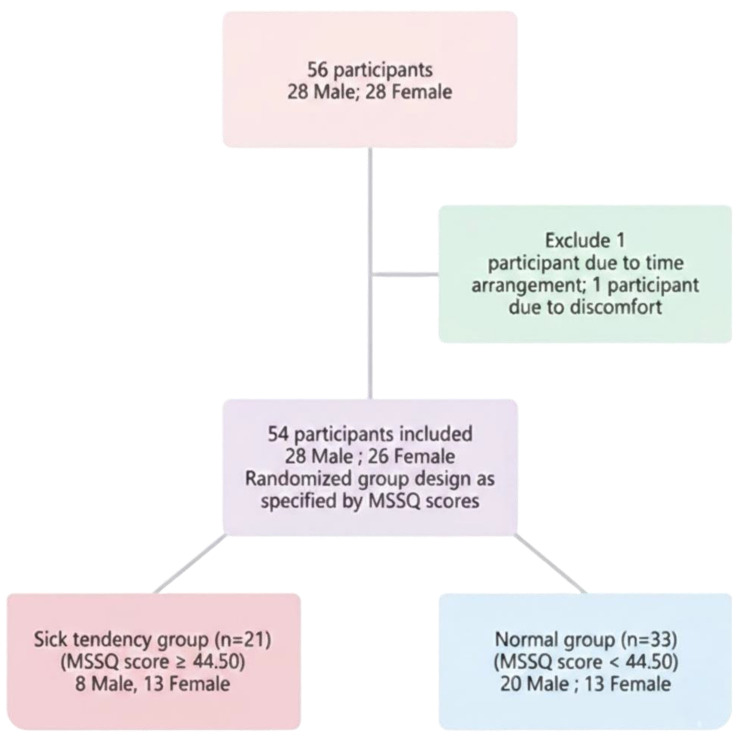
Flowchart of Participant Selection Process.

A total of 56 participants without a history of ocular-related diseases, long-term visual disturbances, imbalance problems, or prolonged use of medications such as sleeping pills or anxiety drugs were recruited for the study. After 2 participants were excluded due to scheduling conflicts and physical discomfort, respectively, 54 participants successfully completed the study (Figure 1). The participants were divided into two groups according to their MSSQ score (cutoff point = 44.5). The demographic data are presented in Table 1. The study participants were divided into two groups depending on their MSSQ scores and VR exposure experiment (53.24 ± 6.17 and 32.67 ± 5.66, t = 10.363, *p* < 0.001): 21 individuals in the sick tendency group and 33 in the normal group. The mean age of the participants was 22.14 ± 1.49 years and 22.39 ± 1.87 years, respectively. Regarding refractive power, the equivalent spherical power of the right eye was −5.40 ± 3.67 D and −4.46 ± 2.98 D; for the left eye, it was −5.23 ± 3.44 D and −4.26 ± 2.98 D. The 95% confidence interval ellipse areas of the center of pressure were 53.69 ± 55.78 and 35.82 ± 32.64 mm^2^, while the centers of pressure sway velocity were 9.02 ± 1.25 and 8.77 ± 1.23 mm/s. Demographic data analysis revealed that the two groups did not significantly differ in age, VA and equivalent spherical power.

### 2.3. Research Materials

#### 2.3.1. MSSQ

Motion sickness susceptibility was subjectively assessed using the Motion Sickness Susceptibility Questionnaire (MSSQ) [13,22,23,24], which demonstrated excellent internal consistency in this study (Cronbach α = 0.877). To ensure the robustness of group assignment, a dual-validation approach combining psychometric scores and experimental exposure was employed. In addition to completing the MSSQ, all participants underwent 20–30 min of VR exposure to validate their susceptibility. A receiver operating characteristic (ROC) curve analysis was conducted to establish an optimal MSSQ cutoff point of 44.5 (AUC = 1.0, both sensitivity and specificity reaching 100%). Participants with MSSQ scores > 44.5 who concurrently reported subjective motion sickness during VR exposure were assigned to the susceptible (ST) group. Conversely, those who did not experience motion sickness and had MSSQ scores ≤ 44.5 were assigned to the normal group.

#### 2.3.2. Binocular Visual Function

Binocular visual function was examined. The View-M Digital Visual Acuity Chart (Quan Chin Industrial Co., Taichung, Taiwan), the Saladin Near Point Balance Card (Bernell Co., Mishawaka, IN, USA), and the Sine Wave Contrast Test (SWCT, Visumetrics Co., Clearwater, FL, USA) were used for visual acuity (logMAR) and contrast sensitivity (cpd) assessment. Computerized refractive errors were measured using a Nidek AR 800 autorefractor (Nidek, Gamagori, Japan), and retinoscopy was conducted for confirmation. Subjective refraction, distance and near fusional convergence and divergence (prism diopter, PD), and distance fixation disparity (PD) were examined using a TOPCON VT-10 phoropter (Topcon, Tokyo, Japan), the Saladin Near Point Balance Card (Bernell Co., Mishawaka, IN, USA), and the TMV Near VisionCard (Brighten Optix Co., Taipei, Taiwan), respectively. The Howell phoria test was performed to evaluate the distance (3 m) and near phoria (40 cm) (DLP and NLP). The Royal Air Force rule (Bernell Co., Mishawaka, IN, USA) was used to measure the near point of convergence (NPC, cm), near point of accommodation (NPA, diopter), and amplitude of accommodation (AA, cycle). The gradient AC/A ratio was calculated by simultaneously placing +1.00 D and −1.00 D lenses on both eyes and measuring the change by each lens in the near eye position. Stereopsis was assessed using the Frisby Stereo Test (Kay Pictures Ltd., Tring, UK), and monocular and binocular accommodative facilities were measured using ±2.00 D flipper lenses (cycle).

#### 2.3.3. Visual Perception

##### Body Balance

For analysis, the mean of the two balance measurements was calculated for each participant to provide a stable estimate of postural stability. A Physio Sensing (Sensing Future Tech., Coimbra, Portugal) foot pressure plate was used to test the static balance. It consists of 1600 pressure sensors measuring 10 mm × 10 mm. It can record pressure maps, center of pressure (COP), body sway, and weight distribution. At the beginning of the test, the participants were instructed to stand in a designated position and focus on a visual stimulus. The test was conducted twice. Each measurement lasted 1 min, and a rest period of 1 h was allotted between the tests. After measurements were taken with the balance foot pressure plate, the following important values were considered: (1). The 95% confidence ellipse area of COP (ellipse of COP, mm^2^) encompasses 95% of the trajectory of the COP movement during the test using the minimum ellipse area. The smaller the area, the greater the stability. (2). The mean velocity of body sway (mm/s) represents the average distance the COP sways during the test. The smaller the sway velocity, the greater the stability.

##### Motor-Free Visual Perception Test (MVPT)

MVPT-4 (Therapro Inc., Framingham, MA, USA) was used for assessments. The test was conducted for approximately 20 min in a quiet environment during the daytime when the participants’ attention was focused. It was composed of 45 items and divided into five dimensions: spatial relationships, visual discrimination, figure-ground, visual closure, and visual memory. Only figure-ground, spatial relationships, and visual closure were used for testing in this study because these three subtests have a higher correlation with spatial perception.

##### Peripheral Awareness (PA) Test

Peripheral awareness was examined using a peripheral awareness chart (Bernell Co., Mishawaka, IN, USA), which is a square measuring 45 cm^2^. It was placed at a distance equivalent to one forearm’s length in front of the participant, ensuring their eyes were level with the center position. At the start of the measurement, the examiner randomly pointed to a target on the chart, and the participants used their dominant hand to indicate the target’s location while recording the number of targets pointed out by the participant within 1 min.

### 2.4. Data and Statistical Analysis

Statistical analyses were conducted using IBM SPSS Statistics v.26 (IBM Corp., Armonk, NY, USA). Study outcomes were pre-specified as primary or exploratory to maintain hypothesis integrity. Continuous variables were compared between groups using independent *t*-tests, while categorical data were analyzed via chi-square tests. To account for multiple comparisons among primary core indicators and secondary clinical measures, *p*-values were adjusted using the False Discovery Rate (FDR) method. For all analyses, effect sizes (Cohen’s d for *t*-tests and Cramér’s V for chi-square) were reported. Statistical significance was defined as a two-tailed *p* < 0.05.

## 3. Results

### 3.1. Motion Sickness and Binocular Visual Functions

The binocular visual function was compared between the ST and normal groups via an independent *t*-test. It was examined in terms of PFV, NFV, PRA, NRA, AF, VF, stereopsis, near and distance FD, NPA, NPC, and contrast sensitivity. For certain test items where participants did not respond (e.g., no blur point) or where the results had directional characteristics (e.g., esophoria or exophoria), data were recorded as nominal or ordinal variables. Accordingly, a statistical comparison was conducted through chi-square analysis. The results of the analysis are shown below.

#### 3.1.1. Distance and near Fusional Vergence

The “break” value represents the ability to maintain binocular fusion, while the “recovery” value indicates the ability to sustain and stabilize binocular fusion. The average values of distance and near fusional vergence in the ST group were higher than those in the normal group (Table 2). Regarding distance fusional vergence ability, the two groups did not significantly differ in NFV break (t = −1.64, *p* = 0.054, d = 0.45) and PFV break (t = −0.59, *p* = 0.264, d = 0.16). However, the groups exhibited significant differences in NFV recovery (t = −1.87, *p* = 0.036, d = 0.52), VFV break (t = −1.81, *p* = 0.038, d = 0.50), and VFV recovery (t = −1.87, *p* = 0.034, d = 0.52). To maximize statistical power while controlling for multiple comparisons, FDR adjustments were applied to a pre-specified set of primary outcomes (distance NFV recovery, VFV break, and VFV recovery; other parameters were treated as exploratory). Following FDR adjustments for these primary clinical measures, all three distance parameters remained statistically significant (q = 0.038).

In terms of near fusional vergence ability (Table 2), the ST and normal groups did not significant vary in NFV break (t = −0.41, *p* = 0.341, d = 0.11), NFV recovery (t = −0.62, *p* = 0.267, d = 0.17), VFV break (t = −1.57, *p* = 0.064, d = 0.44), and VFV recovery (t = −0.94, *p* = 0.176, d = 0.26). Conversely, they significantly differed in PFV break (t = −1.70, *p* = 0.047, d = 0.47), while they nominally differed in PFV break (t = −1.70, *p* = 0.047, d = 0.47) in the initial test, this significance did not survive the FDR adjustment (q = 0.096).

Notably, the observed effect sizes for distance NFV and VFV recovery exceeded 0.50, representing a medium-strength effect. This suggests that the impairment in fusional stabilization in the ST group is not merely a statistical artifact but a robust physiological characteristic.

**Figure 2:**
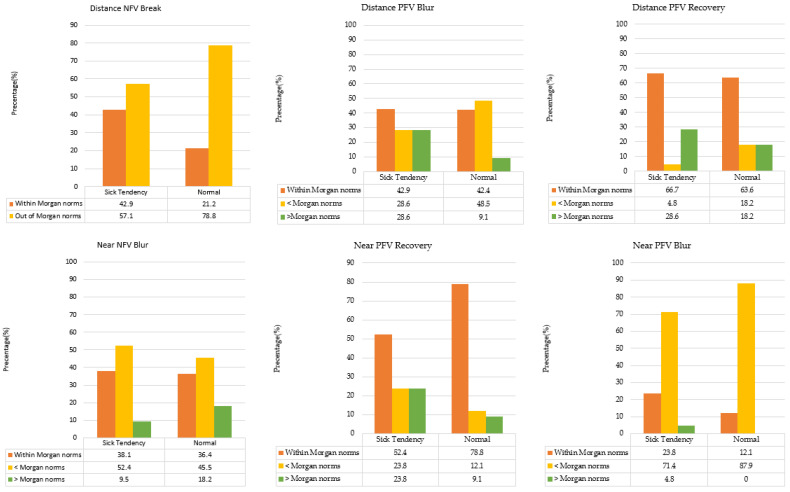
Prevalence in Distance and Near fusional vergence.

Due to the absence of a “blur point” or specific directional characteristics in some participants, subjects were categorized according to Morgan’s norms. Chi-square analysis revealed no statistically significant differences across the tested parameters: D/NFV/break, χ^2^ = 2.884, *p* = 0.089, Cramer’s V = 0.23; D/PFV/blur, χ^2^ = 4.172, *p* = 0.124, Cramer’s V = 0.28; D/PFV/recovery, χ^2^ = 2.424, *p* = 0.298, Cramer’s V = 0.21; N/NFV/blur, χ^2^ = 0.788, *p* = 0.674, Cramer’s V = 0.12; N/PFV/blur, χ^2^ = 3.050, *p* = 0.218, Cramer’s V = 0.24; N/PFV/recovery, χ^2^ = 4.235, *p* = 0.120, Cramer’s V = 0.28. Despite the lack of statistical significance, distinct distributional trends emerged. The ST group exhibited a higher proportion of individuals within Morgan’s norms for distance NFV break and near PFV blur compared to the normal group. Conversely, they showed a lower frequency of normal values for near PFV recovery. These results suggest that individuals susceptible to motion sickness habitually prioritize visual abilities for distant targets, which may lead to a reduced capacity to stabilize binocular fusion during near tasks, particularly when facing balance disruptions (Figure 2).

Although Chi-square analyses were not statistically significant, near-medium effect sizes (Cramér’s V = 0.28) for D/PFV/blur and N/PFV/recovery reveal a systematic deficit in the ST group. This group showed a notable tendency to fall outside Morgan’s norms, particularly regarding near-fusional recovery. Such categorical trends—more sensitive than aggregate means—imply that motion-sickness-susceptible individuals may employ compensatory mechanisms, exerting greater visual effort to maintain image stability during balance disruptions.

#### 3.1.2. Accommodation and Vergence

In terms of accommodative and vergence abilities, the mean values of each variable suggested that the accommodative and vergence functions of the normal group were better than those of the ST group. However, statistical analysis revealed that only NPA (t = −2.06, *p* = 0.022, d = 0.57) significantly differed between the two groups. While raw *p*-values indicated a significant difference in NPA, this did not survive FDR correction for multiple comparisons (q = 0.060). However, interpreting these findings through effect size provides additional insight into the magnitude of these differences. NPA demonstrated a medium effect size, suggesting a clinically relevant reduction in accommodative amplitude in the ST group despite the conservative statistical threshold (Table 3).

Other parameters, such as NRA (t = −0.72, *p* = 0.239, d = 0.20), PRA (t = 0.17, *p* = 0.432, d = 0.05), monocular accommodative facility (t = −1.38, *p* = 0.082, d = 0.38), binocular accommodative facility (t = −0.60, *p* = 0.265, d = 0.17), vergence facility (t = −0.42, *p* = 0.337, d = 0.12), NPC break (t = 1.09, *p* = 0.141, d = 0.30), and NPC recovery (t = 0.52, *p* = 0.354, d = 0.14) did not significantly vary between the two groups.

#### 3.1.3. Stereopsis and Contrast Sensitivity

Stereopsis performance was quantified and reported as log_10_ (arc seconds), where lower values indicate better depth perception performance; Contrast sensitivity values represent the contrast threshold (expressed as a percentage) at which participants could reliably detect sine-wave gratings at each spatial frequency (cpd = cycles per degree). Results showed stereopsis significantly differed (t = −1.69, *p* = 0.048, d = 0.47). However, no significant differences (Table 3) were found in contrast sensitivity at spatial frequencies of 1.5 cpd (t = 0.70, *p* = 0.472, d = 0.19), 3 cpd (t = 0.26, *p* = 0.397, d = 0.07), 6 cpd (t = 0.37, *p* = 0.357, d = 0.10), 12 cpd (t = 0.00, *p* = 0.499, d = 0.00), and 18 cpd (t = −0.02, *p* = 0.491, d = 0.01). These findings indicated that the stereopsis performance of the ST group was poorer than that of the normal group, while contrast sensitivity remained comparable between the two groups across high and low spatial frequencies.

#### 3.1.4. Distance and near Phoria and Fusional Disparity (FD)

**Figure 3:**
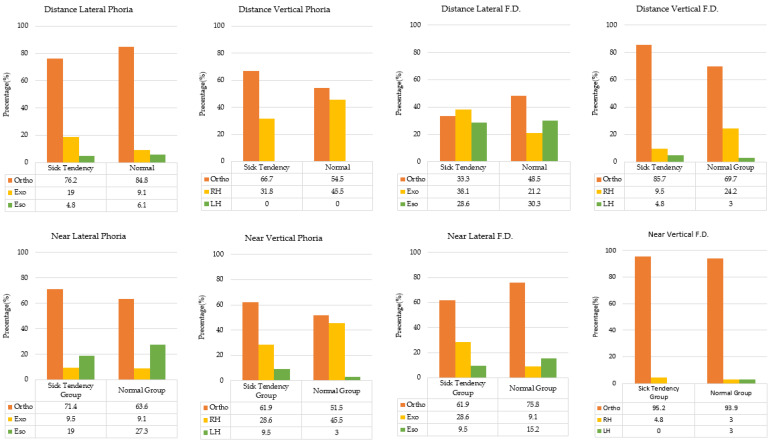
Prevalence in Distance and Near phoria and fixation disparity (FD).

Lateral phoria or lateral FD was classified into three categories (ortho, eso, and exo); vertical phoria or vertical FD was also classified into three categories (ortho, right hyper [RH], and left hyper [LH]) based on Morgan’s norms. Statistical analysis revealed no significant differences between the two groups (Figure 3) in all the phoria variances (distance lateral phoria: χ^2^ = 1.138, *p* = 0.566, Cramer’s V = 0.15; distance vertical phoria: χ^2^ = 0.781, *p* = 0.377, Cramer’s V = 0.12; near lateral phoria: χ^2^ = 0.480, *p* = 0.787, Cramer’s V = 0.09; near vertical phoria: χ^2^ = 2.164, *p* = 0.339, Cramer’s V = 0.20) and all the FD variances (distance lateral F.D.: χ^2^ = 2.022, *p* = 0.364, Cramer’s V = 0.19; distance vertical F.D.: χ^2^ = 1.883, *p* = 0.390, Cramer’s V = 0.19; near lateral F.D.: χ^2^ = 3.586, *p* = 0.166, Cramer’s V = 0.26; and near vertical F.D.: χ^2^ = 0.743, *p* = 0.690, Cramer’s V = 0.12). The proportion of “ortho” in near lateral fixation disparity was higher in the normal group (75.8%), whereas the ST group showed more “exo” (28.6%). Although the Chi-square analysis did not reach statistical significance, the calculated effect size (Cramér’s V = 0.26) indicates a near-medium association between near-lateral FD and motion sickness susceptibility. These findings suggested that clinicians should still consider the potential effect of near lateral fixation disparity on motion sickness susceptibility.

### 3.2. Motion Sickness and Visual Perception

The visual perception items in this study were PA, MVPT (including subcategories such as visual figure-ground, VFG; visual spatial, VS; and visual closure, VC), the ellipse of COP, and the mean velocity of body sway. Independent sample *t*-test analysis revealed that the ellipse of COP (t = 2.03, *p* = 0.047, d = 0.40) significantly differed between the ST and normal groups; furthermore, other variables such as PA (t = −0.73, *p* = 0.223, d = 0.14), MVPT (t = 0.88, *p* = 0.191, d = 0.17), MVPT figure-ground (t = −0.47, *p* = 0.319, d = 0.06), MVPT visual spatial (t = 0.43, *p* = 0.339, d = 0.08), MVPT visual closure (t = −0.10, *p* = 0.459, d = 0.02), and mean velocity of body sway (t = 0.73, *p* = 0.234, d = 0.10) did not significantly vary. Although the ellipse of COP reached statistical significance, its medium effect size further confirms that the difference in postural stability between the ST and normal groups is not only statistically valid but also practically meaningful.

## 4. Discussion

This study demonstrated that the distance NFV (break and recovery), distance VFV (break and blur), and near PFV break point of the motion sickness susceptibility group were better than those of the normal group. Conversely, the normal group showed superior performance in stereopsis and a significantly closer Near Point of Accommodation (NPA) than the ST group. Previous research suggests that morphological asymmetries within the vestibular system can impair the integration of visual and vestibular signals; as a result, compensatory mechanisms may rely more heavily on visual input for sensory integration [25]. Consequently, studies have confirmed that visual signals play a crucial role in modulating the development of motion sickness, suggesting that the effective management of sensory conflicts is essential for alleviating symptoms [2,7,26,27,28,29].

Regarding distance NFV (blur and break), a higher proportion of individuals in the ST group scored within Morgan’s norms. This suggests that these individuals may exert greater accommodative effort during near tasks, potentially contributing to their receded near point of accommodation (NPA). For distance PFV blur, a significantly higher percentage of the ST group performed above Morgan’s norms, while fewer fell below the norms compared to the normal group. This phenomenon indicates that individuals in the ST group tend to utilize excessive accommodative convergence when shifting focus from distance to near targets. However, some studies argue that blur points can be inconsistent during clinical assessments, making them less reliable for objective evaluation [30].

In the near PFV recovery, the percentage of individuals in the normal group that fell within Morgan’s standard values was higher than that in the ST group. This result suggested that the normal group exhibited better and faster autonomous responses to convergence stimuli and to spatial perception information. In terms of fixation disparity and phoria, only the near lateral fixation disparity exhibited a near-significant difference; a higher proportion maintained ortho in the normal group, while the other group performed exo. Under stress, binocular vision likely experiences fixation disparity, indicating a non-compensatory phoria [31]. Therefore, individuals with motion sickness susceptibility might be more prone to the misalignment of visual axes under stress, although they did not experience diplopia.

In other visual function aspects, NPA was poorer in the ST group than in the normal group; this finding indicates a reduced amplitude of accommodation, which may be reflected in the distance NFV blur results. Since the proportion of individuals in the ST group with distance NFV blur was higher than that in the normal group, they likely experienced more accommodative stress during the overcorrection of myopia. Approximately 20% of the NFV blur points of the ST group were below Morgan’s values, whereas only 10% of the NFV blur points of the normal group were below Morgan’s values. However, accommodation is a single ability, while fusional convergence involves a cumulative effect of accommodation and convergence [32,33]. Additionally, the poorer performance in the near point accommodation in the ST group might be related to weaker spatial positioning skills because accommodation contributes to acquiring depth information [34]. The ST group also demonstrated poorer stereopsis; fewer individuals maintained ortho during near horizontal fixation than the normal group, which was also related to spatial positioning. Individuals prone to motion sickness tend to have poorer alignment of fixation and visual axes; consequently, they experience more difficulty in accurately perceiving object positions in three-dimensional space and maintaining postural balance [35,36,37,38,39].

In summary, this study demonstrates that accommodation–vergence conflict—specifically the interplay between convergence, accommodation, stereopsis, and postural stability (center of pressure, COP)—serves as a critical indicator of motion sickness susceptibility. Our findings reveal that distance VFV (break and recovery) and distance NFV recovery differ significantly between the groups. This aligns with previous research suggesting that postural stability can be compromised by vertical prism stimulation [40]. In near tasks, the interaction between accommodation, convergence (specifically near PFV break), and stereopsis is closely linked to motion sickness. These findings underscore that while accommodation and convergence are fundamental oculomotor functions, their proficiency is essential for maintaining effective stereopsis and spatial perception. These high-level visual processes are intrinsically tied to body balance, and their impairment appears to be a key factor in the development of motion sickness [41].

According to the findings of this study, it has significant clinical implications, particularly in the areas of vision therapy/rehabilitation. Targeted Vision Therapy (VT) aimed at expanding fusional vergence ranges and accommodative amplitude may reduce symptoms by resolving sensory-vergence conflicts and enhancing postural stability. Indicators such as distance VFV, near PFV break, and stereopsis showed significant group differences and warrant further investigation as potential clinical indicators, though prospective validation would be needed before clinical implementation.

## 5. Conclusions

Accommodation and convergence are fundamental oculomotor functions that interact synergistically to facilitate binocular fusion, ultimately leading to the formation of stereopsis. As the highest expression of binocular function, stereopsis enables precise spatial localization within three-dimensional environments. Throughout this functional hierarchy, spatial positioning abilities are progressively refined. The superior fusion and convergence capabilities observed in the motion sickness susceptibility (ST) group may serve as a compensatory mechanism for their underlying deficits in accommodation, lateral fixation disparity, and stereopsis.

**Figure 4:**
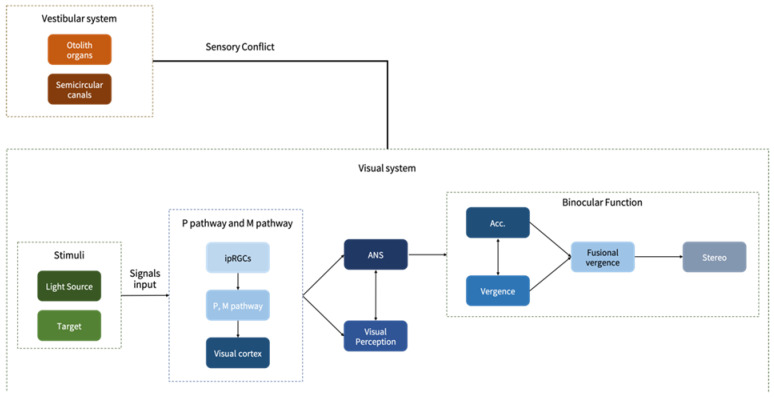
The relationship between motion sickness, binocular vision and visual perception.

The vestibular system, comprising the otolith organs and semicircular canals, interacts with the visual system; when these inputs conflict, motion sickness occurs. The visual system processes light stimuli and fixation targets via intrinsically photosensitive retinal ganglion cells (ipRGCs), transmitting signals through the Parvocellular (P) and Magnocellular (M) pathways to the visual cortex [42,43]. Driven by the autonomic nervous system (ANS), this interaction modulates accommodation and vergence, synergistically influencing binocular fusion and high-level functions such as depth and visual perception. Optimal visual perception relies on the healthy functioning of both the M and P pathways [44,45]. This sensory conflict between the vestibular and visual systems, which serves as the foundation for our exploration of visual factors in motion sickness, is illustrated in Figure 4.

## Figures and Tables

**Table 1:** Basic information of the participants, M (SD).

	Sick Tendency N = 21	Normal N = 33	Total N = 54	T	*p*
Age	22.14 (1.49)	22.39 (1.87)	22.30 (1.72)	−0.520	0.606
MSSQ Scores	53.24 (6.17)	32.67 (5.66)	42.87 (10.17)	10.363 *	<0.001
OD Spherical Equivalent (Diopter)	−5.40 (3.67)	−4.46 (2.96)	−4.83 (3.26)	−1.039	0.304
OS Spherical Equivalent (Diopter)	−5.23 (3.44)	−4.26 (2.98)	−4.64 (3.17)	−1.091	0.280
Monocular VA at Distance (log MAR)	0.01 (0.05)	0.02 (0.06)	0.02 (0.06)	−0.346	0.731
Binocular VA at Distance (log MAR)	−0.05 (0.05)	−0.03 (0.07)	−0.04 (0.06)	−0.872	0.386
Monocular VA at Near (log MAR)	0.01 (0.02)	0.02 (0.02)	0.01 (0.02)	−0.889	0.378
Binocular VA at Near (log MAR)	0.01 (0.02)	0.01 (0.01)	0.01 (0.01)	−0.387	0.705

* *p* < 0.05.

**Table 2:** Difference in Distance and Near fusional vergence (prism diopter) between the two groups.

Distance Fusional Vergence	Sick Tendency N = 21	Normal N = 33	t	P FDR q	Cohen’s d	Near Fusional Vergence	Sick Tendency N = 21	Normal N = 33	t	P FDR q	Cohen’s d
Distance NFV break (PD)	10.38 (4.07)	8.85 (2.80)	−1.64	0.054	0.45	Near NFV Break (PD)	19.10 (6.55)	18.39 (5.71)	−0.41	0.341	0.11
Distance NFV recovery (PD)	6.24 (3.17)	4.91 (2.10)	−1.87 *	0.036 0.038	0.52	Near NFV Recovery (PD)	14.81 (5.99)	13.82 (5.49)	−0.62	0.267	0.17
Distance PFV break (PD)	21.86 (5.48)	19.65 (6.89)	−0.59	0.264	0.16	Near PFV Break (PD)	21.24 (7.00)	18.00 (6.05)	−1.70 *	0.047 0.096	0.47
Distance VFV break (PD)	2.88 (1.00)	2.38 (1.00)	−1.81 *	0.038 0.038	0.50	Near VFV Break (PD)	3.52 (1.26)	2.95 (1.35)	−1.57 *	0.064 0.096	0.44
Distance VFV recovery (PD)	1.45 (0.81)	1.11 (0.56)	−1.87 *	0.034 0.038	0.52	Near VFV recovery (PD)	1.90 (1.10)	1.64 (0.98)	−0.94	0.176 0.176	0.26

* *p* < 0.05.

**Table 3:** Difference in accommodation, vergence, stereopsis, and contrast sensitivity between the two groups.

Accommodation	Sick Tendency N = 21	Normal N = 33	t	P FDR q	Cohen’s d	Contrast Sensitivity	Sick Tendency N = 21	Normal N = 33	t	*p*	Cohen’s d
NRA (Diopter)	1.96 (0.59)	1.83 (0.70)	−0.72	0.239	0.20	Contrast Sensitivity (1.5 cpd)	86.57 (31.53)	87.24 (35.84)	0.70	0.472	0.19
PRA(Diopter)	−2.46 (1.44)	−2.38 (1.97)	0.17	0.432	0.05	Contrast Sensitivity (3 cpd)	103.43 (41.00)	106.42 (40.47)	0.26	0.397	0.07
Monocular Accommodative Facility (Cycle)	14.71 (4.90)	13.12 (3.57)	−1.38	0.082 0.123	0.38	Contrast Sensitivity (6 cpd)	129.05 (38.43)	133.18 (40.98)	0.37	0.357	0.10
Binocular Accommodative Facility (Cycle)	14.86 (3.94)	14.06 (5.18)	−0.60	0.265	0.17	Contrast Sensitivity (12 cpd)	73.33 (34.33)	73.30 (30.17)	0.00	0.499	0.00
NPA (Diopter)	6.41 (3.01)	5.18 (1.29)	−2.06 *	0.022 0.060	0.57	Contrast Sensitivity (18 cpd)	36.90 (19.71)	36.76 (23.15)	−0.02	0.491	0.01
**Vergence**	**Sick** **Tendency** **N = 21**	**Normal** **N = 33**	**t**	***p***	**Cohen’s d**	**Stereopsis**	**Sick** **Tendency** **N = 21**	**Normal** **N = 33**	**t**	***p***	**Cohen’s d**
Vergence Facility (Cycle)	14.67 (3.40)	14.18 (4.48)	−0.42	0.337	0.12	Stereopsis log_10_ (arc seconds)	1.26 (0.18)	1.20 (0.57)	−1.69 *	0.048	0.47
NPC break (cm)	1.91 (2.49)	3.35 (5.72)	1.09	0.141	0.30						
NPC recovery (cm)	2.48 (3.28)	3.14 (5.22)	0.52	0.354	0.14						

* *p* < 0.05.

## Data Availability

All data generated or analyzed during this study are included in this published article. Correspondence and requests for materials should be addressed to C.-Y.C.
